# Ion-Pair Mediated Valence Isomerization of Selected Cyclic C_7_H_8_ Molecules Trapped in Insertion Complexes

**DOI:** 10.3390/ijms27073086

**Published:** 2026-03-28

**Authors:** Chen Liang, Fedor Y. Naumkin

**Affiliations:** Faculty of Science, Ontario Tech University, Oshawa, ON L1G 0C5, Canada; chen.liang@ontariotechu.ca

**Keywords:** intermolecular interactions, insertion complex, tautomerization, ion-pair, ab initio calculations, norcaradiene, cycloheptatriene

## Abstract

Highly polar M-mol-X (M = alkali metal, mol = molecule, X = halogen) insertion complexes have been predicted to offer potential practical applications, including molecular interactions with light, ion-pair induced isomerization, etc. In the present work, the insertion complexes of the seven-membered, fused bicyclic norcaradiene and its monocyclic isomer trapped in Li-I, Na-I, and K-I counterion pairs were investigated using ab initio methods. The structures, stability, polarities, and simulated infrared spectra are analyzed and the effects of the insertion on the norcaradiene to cycloheptatriene isomerization process are examined. Furthermore, an uncommon bond between iodine and a fully substituted carbon atom is reported upon and hypothesized to be catalyzed by the presence of the cation in the insertion complexes.

## 1. Introduction

**Figure 1:**
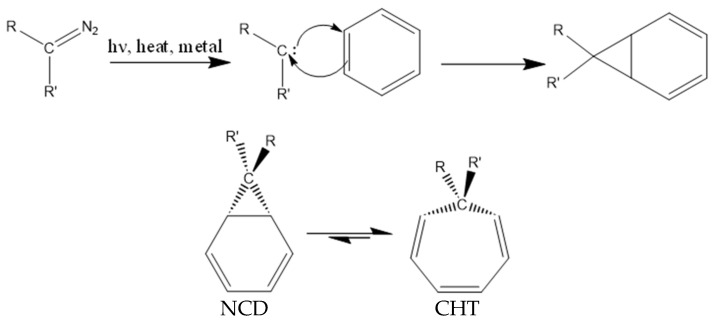
Büchner ring expansion reaction mechanism.

The Büchner ring expansion reaction is a method used to synthesize seven membered carbon rings [1]. The mechanism of the Büchner ring expansion reaction is shown in Figure 1. The second step of the expansion involves an isomerization process called valence tautomerization, where the bond shared between the cyclopropane and cyclohexadiene rings of an NCD (norcaradiene) molecule or derivative breaks to yield a CHT (cycloheptatriene) molecule or derivative [2]. Efforts have been made to improve the efficiency, selectivity and scalability of the Büchner ring expansion reaction through a better understanding of the NCD ↔ CHT equilibrium and rearrangement process [3,4]. The equilibrium for the unsubstituted molecules favors the CHT product over NCD, as CHT is more thermodynamically stable due to the high structural strain of the cyclopropane group in NCD [3,5]. Substitutions, catalysts, and other reaction conditions can shift the equilibrium towards either side, improving product stability [6].

Molecules inserted between alkali halide counterions have been predicted to have higher dipole moments than their free alkali halide counterparts due to the increased charge separation present when the counterions are held apart by the inserted molecule [7,8]. These M-mol-X (M = alkali, mol = molecule, X = halide) complexes allow numerous possible applications including solar cells [9,10], lasers [10], nonlinear optics [11], ferroelectrics [12], and those based on molecular interactions, such as complexation, self-assembly, and facilitated chemical reactions [7,8]. Notably, a catalytic effect was observed for the cubane to ladderene isomerization in the Li-C_8_H_8_-F insertion complex [7], whereas an opposite, inhibiting effect was found for the benzene trioxide to trioxonine isomerization in the Cs-C_6_H_6_O_3_-I insertion complex [8]. This suggests that the internal field generated by the counterions of an insertion complex can be leveraged to control the isomerization of the inserted molecule.

Recent studies have computationally shown the successful trapping of various molecules in between alkali–halide pairs, ranging from cubane [7] to unsubstituted [13,14,15], oxygenated [8], and fluorinated cyclic hydrocarbons [16,17,18]. Many of these molecules are similarly sized and structured as NCD and CHT, suggesting a high likelihood that these two molecules can successfully host counterion pairs in insertion complexes.

Typically, electrostatic attraction between the counterions and an inserted polar molecule plays a key role in their mutual hosting properties within insertion complexes [8]. However, non-polar molecules with a concave electron density can also successfully host a counterion pair [7]. This concave shape stabilizes the insertion complex by creating a cavity that confines the ion, preventing any movement which may cause the ions to recombine [7]. Recombination of the counterions breaks the insertion complex and can occur when either ion moves around the molecule or when the ions penetrate the center of the molecule and pass through its cyclic opening [8]. The latter recombination pathway is particularly significant as the tunneling of ions through cyclic molecules can provide insights on the behavior of ions passing through planar membranes consisting of similar cyclic structures [15].

In the present work, we consider complexes of NCD and CHT trapped between various cations (of Li, Na, and K) and the I anion to investigate the effects of the pressure exerted by the mutually attracting counterions and the internal field generated in the insertion complexes on the process of NCD rearrangement into CHT. Additionally, we determine the energy barriers for cation penetration through the seven-membered CHT ring to assess the potential for ion permeability in similarly structured planar membranes. Furthermore, the predicted infrared spectra of the various complexes are provided to support possible future experiments with these insertion complexes.

## 2. Results and Discussion

### 2.1. Structures and Stabilities

**Figure 2:**
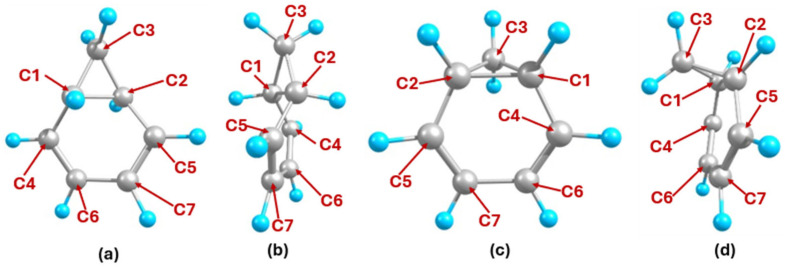
Optimized geometries of e-NCD: (**a**) front view and (**b**) side view and a-NCD: (**c**) front view and (**d**) side view.

Figure 2 shows the two optimized conformers of NCD obtained in this work, differing by the orientation of the cyclopropane ring consisting of the C1, C2, and C3 atoms. The structure where this cyclopropane ring extends in an equatorial direction, termed as e-NCD (equatorial-NCD), features a non-planar cyclohexadiene ring. The structure where the cyclopropane ring extends in an axial direction, termed a-NCD (axial-NCD), features a flat, less strained cyclohexadiene ring. As a result, the a-NCD conformer exhibits a much higher stability when compared to the e-NCD conformer, being 1.73 eV lower in energy. Therefore, subsequent calculations and references of NCD will refer to the a-NCD conformer.

**Figure 3:**
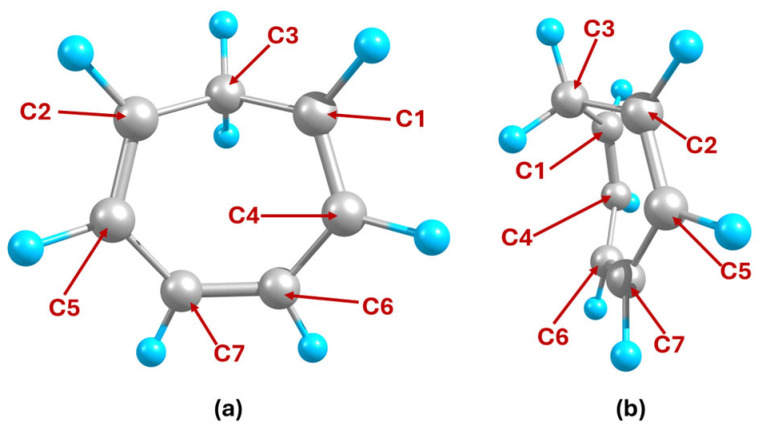
Optimized geometries of CHT: (**a**) front view and (**b**) side view.

Figure 3 shows the optimized structure of CHT. The absence of the highly strained cyclopropane group makes CHT more stable than NCD by 0.24 eV in accordance with the NCD↔ CHT equilibrium, which favors the CHT side [5].

Table 1 compares the calculated geometry parameters for the two optimized NCD structures with values reported from other literature and Table 2 compares the calculated geometry parameters for the optimized CHT structure with both calculated and experimental values from other literature [19,20]. All the listed geometry parameters differed by less than 5%, suggesting good consistency between the optimizations performed in this study and those found in previous works.

**Figure 4:**
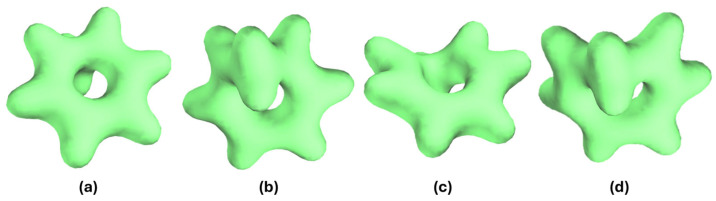
Calculated electron densities of NCD (**a**) front view and (**b**) back view and CHT (**c**) front view and (**d**) back view, where front view is the side without the protruding CH_2_ group and isosurface is at 0.08 a.u.

Figure 4a shows the electron density of NCD, which is concave on the side without the protruding CH_2_ group, promoting cation attachment. Figure 4b shows the electron density of the other side of the NCD molecule. In this region, the anion is stabilized by the δ^+^ charge of the H atom (belonging to the protruding CH_2_ group), which points toward the center of the molecule. A similar electron density distribution is observed for the CHT molecule as seen in Figure 4c,d, where the concave shape is more pronounced due to the larger ring opening. This suggests that similar ion arrangements can occur for the insertion complexes of CHT and NCD, but with stronger ion attachments in the CHT complexes.

The Li, Na, and K atoms were considered for NCD and CHT insertion complexes to determine the effects of ion sizes on isomerization, where smaller ions can approach the molecule more closely. The I atom was chosen because the halogen atom in these insertion complexes is attracted to the H atom attached to the protruding CH_2_ group. Stronger halogens, such as F and Cl could potentially react with this molecule by removing this H atom, thereby breaking the insertion complex.

Table 3 describes the calculated M-mol-I insertion complexes (M = Li, Na, K, mol = NCD, CHT) and the binary (Na-mol)^+^ and (I-mol)^−^ structures. All the insertion complexes were found to be stable, with energies ranging from approximately 3.56 eV to 5.93 eV lower than that of their fragments (M^+^ + mol + I^−^). The stability of the complexes increases as the cation size decreases, from K to Li. This trend is likely due to the smaller cations approaching the molecule more closely [8]. This is supported by the decreasing R_e_(M-C5) and R_e_(M-I) distances as the cation size reduces. Notably, the distance between the ions, R_e_(M–I), has an inverse relationship with the dissociation energy, with a smaller distance between the ions corresponding to a higher dissociation energy of the complex.

**Figure 5:**
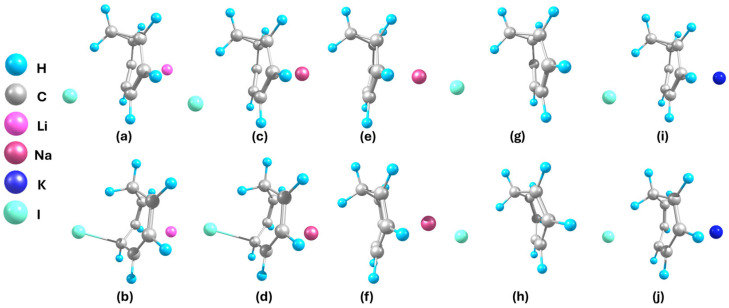
Optimized geometries of (**a**) Li-NCD-I, (**b**) Li-CHT-I, (**c**) Na-NCD-I, (**d**) Na-CHT-I, (**e**) Na-NCD, (**f**) Na-CHT, (**g**) NCD-I, (**h**) CHT-I, (**i**) K-NCD-I, and (**j**) K-CHT-I.

Larger dipole moments are generally observed for greater Re(M-I) distances, as expected. However, the Li and Na based insertion complexes of CHT deviate from this trend. This anomaly is primarily due to a bond which forms between the I anion and the nearest C atom in Li-CHT-I (Figure 5b) and Na-CHT-I (Figure 5d). This interaction reduces the negative charge on the I anion, resulting in the lower dipole moments observed for those complexes. Further details of this bond formation are provided in Section 2.5. Cation-Induced C-I Bonding. For the insertion complexes, the dipole moments do not increase as significantly as the charge separation does. For instance, the dipole moment is only 1.75 times higher in Li-NCD-I when compared to the free LiI diatom (see Section 3 below), despite the charge separation being 2.02 times larger. The same can be seen for the Na-NCD-I complex, where the dipole moment is 1.65 times greater than for the NaI diatom, while the charge separation is 1.93 times greater. This discrepancy can be attributed to the interactions between the ions and the inserted molecule, which redistributes charge density towards the molecule and away from the ions, resulting in a more centralized charge distribution.

The binary ion-molecule complexes exhibit relatively weak stabilities (Table 3). For both the NCD and CHT cases, the Na cation attaches more strongly to the molecule, with attachment energies of 1.30 eV and 1.32 eV, respectively, compared to the attachment energies of the I anion, at 0.39 eV and 0.40 eV. This difference can be attributed to the different shapes of the electron densities at the attachment sites. The cation attachment side has a concave electron density, which stabilizes the ion. Additionally, the position of the ions also contributes to this stronger attachment. In both the NCD and CHT complexes, the cation is positioned near center of the molecule (Figure 5), allowing it to attract more effectively to the molecule’s negatively charged C atoms and double bonds. In contrast, the I anion is repelled by the negatively charged C atoms, limiting its attractive interactions solely to the protruding positively charged H atom attached to C3. Furthermore, the smaller size of the Na cation allows it to approach the molecule more closely compared to the larger I anion.

**Figure 6:**
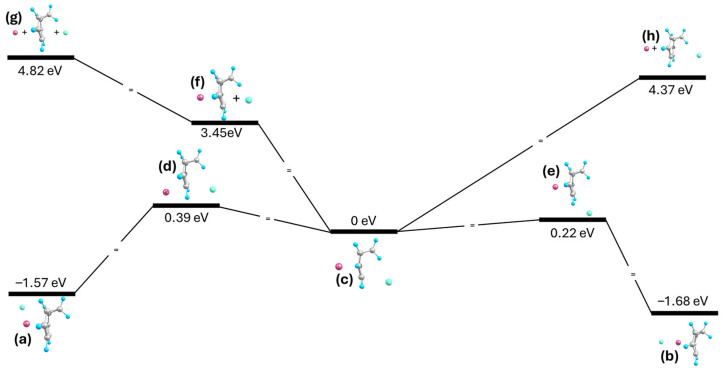
Energy diagram for formation and rearrangement of (**c**) Na-NCD-I, where (**a**) I-Na-NCD results from the (**d**) transition structure, (**b**) I-Na-CHT results from the (**e**) transition structure, and (**f**–**h**) are partially and fully dissociated systems; energy levels not to scale.

The formation of Na-NCD-I can occur via the stepwise attachment of the ions. First, either ion approaches the NCD molecule to form the binary NCD-ion complexes (Figure 6h). Once formed, the other ion approaching from the opposite side (Figure 6f,g) will readily form the insertion complex as the insertion complex is predicted to be more stable by 3.45 eV when compared to (Na-NCD)^+^ + I^−^ and by 4.37 eV when compared to Na^+^ + (NCD-I)^−^, effectively trapping the molecule in between the counterions. The Na-NCD-I insertion complex breaks when the counterions recombine, resulting in the molecule-NaI conformers of the complex. Two recombination pathways were considered: the first pathway involves the Na cation migrating around the bond formed by the C6 and C7 atoms, during which the unrestrained I anion is pulled towards it, forming the transition structure shown in Figure 6d. Afterwards, the Na cation was found to return to its original position, pulling the I anion along with it, resulting in the I-Na-NCD complex shown in Figure 6a. The second pathway involves the I anion migrating around the same C6-C7 bond and towards the Na cation. The Na cation remains in its original position as the I anion approaches it, forming the transition structure shown in Figure 6e. As the I anion continues to move around the NCD molecule, the NCD molecule rearranges into its CHT tautomer, ultimately resulting in the I-Na-CHT complex shown in Figure 6b. These two processes have energy barriers of 0.39 eV and 0.22 eV, respectively, which define the metastability of the Na-NCD-I complex relative to MX-NCD/CHT.

**Figure 7:**
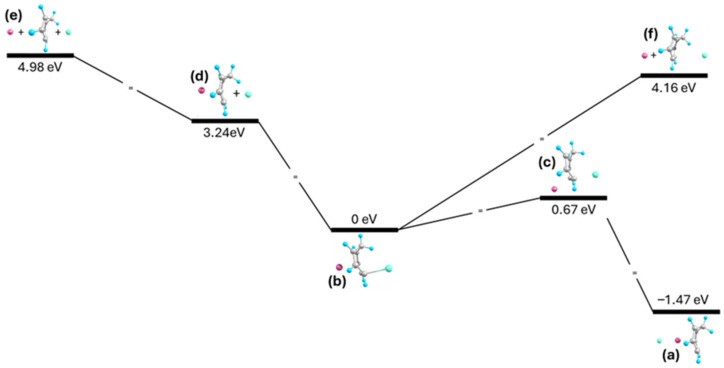
Energy diagram for formation and rearrangement of (**b**) Na-CHT-I, where (**a**) I-Na-CHT results from the (**c**) transition structure, and (**d**–**f**) are partially and fully dissociated systems; energy levels not to scale.

A similar formation process can occur for the formation of the Na-CHT-I insertion complex, where the first step involves attachment of either ion to the molecule (Figure 7f). The remaining ion then attaches, forming the insertion complex. The attachment of the I anion to (Na-CHT)^+^ stabilizes the complex by 3.24 eV and attachment of the Na cation to (CHT-I)^−^ stabilizes the complex by 4.16 eV (Figure 7d,e). For the Na-CHT-I insertion complex, only one recombination pathway was considered. Since the I anion is bonded to the CHT molecule in this insertion complex, it is unlikely to move. Thus, the only likely pathway involves the Na cation moving around the C6-C7 bond, forming the transition structure shown in Figure 7c. As the Na cation approaches the I anion, the C-I bond breaks and the Na cation snaps back to its original position, with the I anion following it. This results in the I-Na-CHT complex shown in Figure 7a. This process has an energy barrier of 0.67 eV, significantly higher than both recombination processes for the Na-NCD-I complex. One contributing factor to the higher energy barrier is the C-I bond, which must break for the counterions to recombine. Additionally, the larger ring opening of the CHT molecule enables the ions to approach more closely, evident by the smaller R_e_ (Na-I) distance in then Na-CHT-I complex when compared to the Na-NCD-I complex. The closer approach of the ions makes them less likely to recombine around the molecule.

### 2.2. Tautomerization

**Figure 8:**
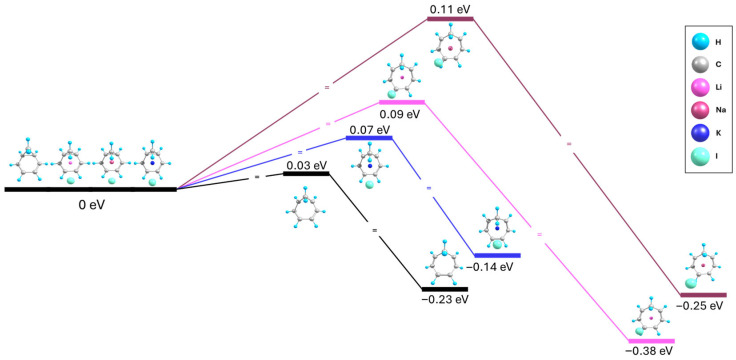
NCD-CHT tautomerization energy diagram for the free molecule, (pink) Li-I insertion complex, (red) Na-I insertion complex, and (blue) K-I insertion complex; energy levels not to scale.

NCD can rearrange into CHT through a process known as valence tautomerization, in which the C1-C2 bond in NCD is broken. This process is accompanied by the change in hybridization of the C1 and C2 carbons from ~sp^3^ to ~sp^2^. The existing double bonds shift from C4=C6 and C5=C7 to C1=C4 and C2=C5 and a new double bond is formed between the C6 and C7 atoms. This process was calculated to have an energy barrier of 0.03 eV and the C1-C2 distance was found to increase by 0.77 Å, from 1.6 Å in NCD to 2.4 Å in CHT. The tautomerization barriers of the free molecules are compared to the trapped molecules in Figure 8.

For the free molecules and the insertion complexes, a single channel of tautomerization was considered, where the C1-C2 bond is broken, and the double bonds rearrange simultaneously. The attraction of the ions exerts pressure on the molecule, pushing the carbon atoms of the ring away from the center. This effect was expected to cause an increase in the C-C bond lengths of the trapped molecule, including the C1-C2 bond in the NCD insertion complex. It was hypothesized that this would catalyze the tautomerization process by reducing the remaining length which the C1-C2 bond needs to stretch when rearranging from NCD to CHT. However, the opposite was observed, and the tautomerization process was inhibited in the insertion complexes. The simplest explanation here is that the same C1-C2 stretching is also observed in the CHT insertion complex, canceling out the effects of the initial C1-C2 lengthening in the NCD insertion complexes. This is evident in the Li-C_7_H_8_-I complex, where the C1-C2 distances increase by roughly the same amount for both the NCD and CHT complexes when compared to their free molecule counterparts (Table 4).

Interestingly, for the Na-NCD-I and K-NCD-I insertion complexes, the C1-C2 distance is slightly decreased. From the binary (Na-NCD)^+^ and (NCD-I)^−^ complexes, it was observed that the addition of the Na cation slightly increases the C1-C2 distance, while the addition of the I anion slightly decreases the distance. This suggests that there are different effects of the cation and anion on the molecule, likely due to withdrawal or donation of electron density to the C1-C2 bond, respectively. For Li-NCD-I, the cationic effect was able to increase the C1-C2 distance, as the smaller Li cation could approach the NCD molecule more closely compared to the larger Na and K cations, whose complexes were relatively more affected by the anionic effect that reduced the C1-C2 distance. The C1-C2 distance was increased in all three of the CHT insertion complexes, where the larger ring opening of the CHT molecule allows for the larger cations to approach more closely.

The largest inhibition effect for the NCD → CHT tautomerization is observed for the Na-C_7_H_8_-I complex, where the energy barrier increases to 0.11 eV. During tautomerization, the C1-C2 was stretched by 0.84 Å, from 1.60 Å to 2.44 Å, marking the largest stretch observed amongst all the complexes. While the larger C1-C2 stretching, which is primarily driven by the attractive forces of the ions, appears to play a role in the inhibition of the tautomerization process, it is not the sole factor. For instance, although the C1-C2 stretching during tautomerization is nearly identical for the Li-C_7_H_8_-I complex when compared to the free molecule, the energy barrier for the Li-C_7_H_8_-I complex is approximately three times greater. The K-C_7_H_8_-I complex exhibits the weakest inhibition effect on the NCD → CHT tautomerization energy barrier. This suggests that the field generated by the ions also plays a role in inhibiting the reaction.

Although the addition of ion-pair increases the barrier relative to that for the free molecule, the relation between cation size and inhibition effect is not immediately apparent. The K-C_7_H_8_-I complex results in the smallest inhibition of the tautomerization process, while the energy barrier increases from the Li-C_7_H_8_-I case to the Na-C_7_H_8_-I case. The K-C_7_H_8_-I case could be distinct due to the unique structure of the K-CHT-I complex. For both the Li-CHT-I and Na-CHT-I complexes, the previously mentioned C-I bond is formed. During the formation of this C-I bond, the bonded C atom undergoes a transition from ~sp^2^ to ~sp^3^ hybridization to accommodate for the new bond to the I anion. This additional process likely raises the energy barrier of the tautomerization process by preventing the formation of the third C=C double bond, thus explaining why the K-C_7_H_8_-I complex exhibits less of an inhibition effect.

The Na-C_7_H_8_-I complex was the most effective at inhibiting the NCD to CHT tautomerization, increasing the barrier by a factor of 3.7, compared factors of 3 and 2.3 for the Li-C_7_H_8_-I and K-C_7_H_8_-I complexes, respectively. The trapped CHT complexes had different stabilities relative to their NCD counterparts. While the free CHT molecule is 0.23 eV more stable than the NCD molecule, the addition of the ions further stabilized the CHT system. The larger ring opening of CHT allows for the ions to approach each other more closely and thereby lowering the total energy of the system. This is evident with Na-CHT-I being 0.25 eV more stable than Na-NCD-I and Li-CHT-I being a remarkable 0.38 eV more stable than Li-NCD-I. However, this relatively increased stability is not observed in the K-CHT-I case, where K-CHT-I is only 0.14 eV more stable than K-NCD-I. This can be attributed to the larger size of the K cation, which prevents it from approaching the molecule as closely as the smaller Na and Li cations. This is supported by the larger K-I distance observed in K-CHT-I compared to K-NCD-I (Table 3). The increased charge separation destabilizes the K-CHT-I structure relative to the other CHT insertion complexes with smaller cations. Another contributing factor is the formation of the C-I bond in the Li-CHT-I and Na-CHT-I complexes, which helps stabilize the complexes. This bond is not present in the K-CHT-I complex.

For the reverse, CHT → NCD tautomerization process, the Li-C_7_H_8_-I and Na-C_7_H_8_-I insertion complexes increased the energy barrier by 0.26 eV and 0.10 eV, corresponding to factors of 1.8 and 1.4, respectively. In contrast, the K-C_7_H_8_-I complex has a catalytic effect on the reverse process, lowering the energy barrier by 0.05 eV, likely due to the lower relative stability of the K-CHT-I complex. This means that for all three insertion complexes, the NCD ↔ CHT equilibrium would shift towards the NCD side when compared to the free molecule. For Li-C_7_H_8_-I and Na-C_7_H_8_-I, this occurs because relative to the energy barriers of the free molecule, forward barrier increased more than the reversed barrier (3 and 3.7 times for the forward barrier versus 1.8 and 1.4 times for the reverse barrier for Li-C_7_H_8_-I and Na-C_7_H_8_-I). In the case of K-C_7_H_8_-I, the forward barrier was increased, while the reverse barrier was decreased. The Na-C_7_H_8_-I insertion complex is the most effective at inhibiting the forward reaction while the Li-C_7_H_8_-I complex is the most effective at inhibiting the reverse reaction. The K-C_7_H_8_-I complex would be the most effective for shifting the overall NCD ↔ CHT equilibrium towards the NCD side.

### 2.3. Infrared Analysis

**Figure 9:**
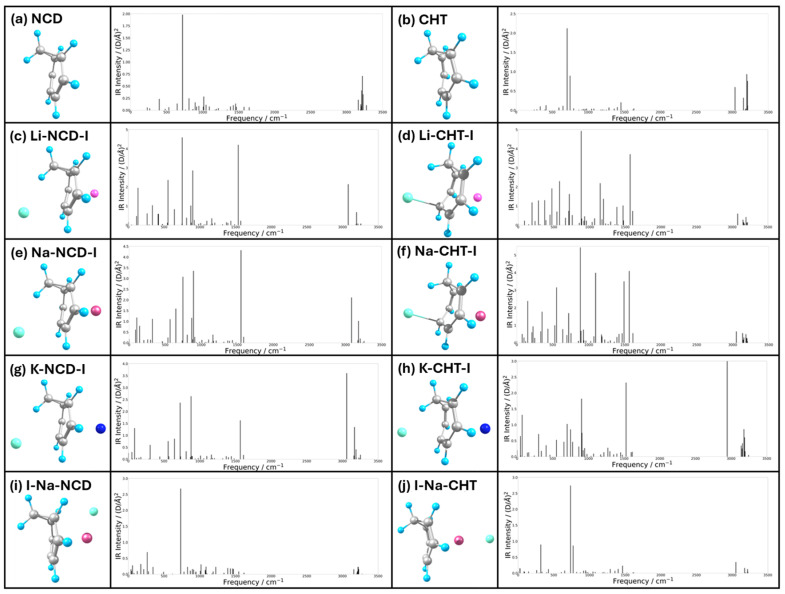
Predicted IR spectra of free NCD, CHT, and their complexes.

The predicted infrared spectra of NCD, CHT, the M-mol-I (M = Li, Na, K and mol = NCD, CHT) insertion complexes, and the I-Na-mol complexes are depicted in Figure 9; the corresponding numerical frequencies and intensities are provided in Section S2 of the Electronic Supplementary Information (ESI). The absence of imaginary frequencies confirms that these structures were successfully optimized to their respective energy minima. The peaks of the spectra of the complexes are more intense than for their free molecule counterparts, likely due to the increased polarity from the ion-pair interactions. For the NCD and CHT molecules (Figure 9a,b), the peaks in the 3000–3300 cm^−1^ range correspond to C-H stretching and are in good agreement with values found in the literature [19]. The brightest peak at 721 cm^−1^ in the NCD spectrum corresponds to C-H out of plane (oop) bending. The CHT spectrum instead shows two peaks for C-H oop bending, at 707 cm^−1^ and 746 cm^−1^. This difference arises because the more planar structure of NCD results in the C-H oop bending to be mostly perpendicular to the plane, whereas the less planar geometry of CHT allows for a greater variety of CH oop bending modes.

The spectra of all the insertion complexes introduced new peaks at around the 1500 cm^−1^ region, which can be attributed to C=C stretching. The ions in the insertion complexes cause an increase in dipole moment, allowing the C=C stretching in the ring to generate net dipole moments in the trapped molecules. The absence of these peaks around the 1500 cm^−1^ region in the I-Na-NCD and I-Na-CHT complexes suggests that they can serve as reliable indicators of insertion complex formation in future experimentation. Additionally, the unique fingerprint region of the predicted spectra of the insertion complexes provides further evidence for confirming their formation.

### 2.4. Ion Penetration

The recombination of the ions through the center of the molecule provides insights into the ion permeability of planar membranes composed of analogous structures by offering an estimated theoretical upper bound of permeability. Here, the Li and Na cations were tested to see if they could pass through the seven membered CHT ring. Penetration of NCD was not considered due to its smaller ring opening and the high likelihood of the C1-C2 bond breaking during the process. The K cation and I anion were not considered for penetration due to their large sizes.

**Figure 10:**
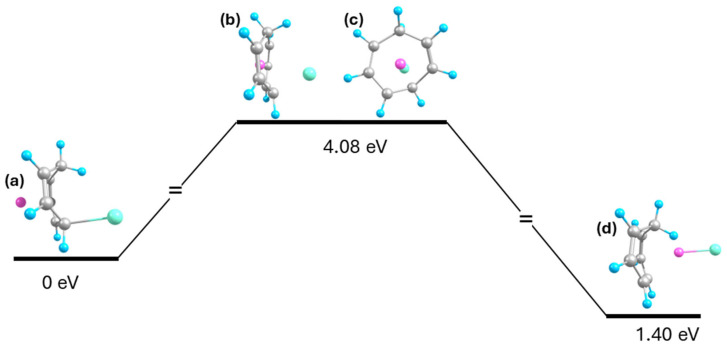
Energy diagram of Li^+^ penetration through the ring of the CHT molecule, where (**a**) is Li-CHT-I, (**b**,**c**) are the side and front views of the transition structure, and (**d**) is the resulting CHT-Li-I structure.

Figure 10 shows the Li cation penetration energy barrier, calculated to be 4.08 eV, suggesting that while ion permeability of membranes with openings similar in size to the CHT ring is theoretically possible, it is statistically negligible under ambient conditions. Na penetration testing revealed that it is even less likely, with the energy barrier exceeding 5 eV even before successful penetration. Consequently, this pathway was suspended as the penetration barrier is significantly higher than the 0.67 eV barrier (Figure 7) for ion recombination around the molecule, suggesting that for the CHT molecule, the ions are much more likely to recombine around it rather than via the direct path through the center. However, this does not imply that membranes consisting of rings similarly sized as CHT would be completely impossible for the Na cation to penetrate. In such membranes, ions have no alternative path around the molecule, leaving the pathway through the ring opening as the only viable option for crossing, although this could only occur under extreme conditions. The calculated energy barriers serve as an indicator of ion permeability, where lower energy barriers suggest higher permeability. These cation penetration barriers are also influenced by the I anion, as the electrostatic attraction between the ions helps pull the cation through the center of the ring. Without the assistance of the anion, the penetration barrier would likely have been higher. These calculated energy barriers represent the theoretical upper limit of permeability because isolated cyclic molecules are less rigid and are more likely to undergo structural deformation than those within a membrane.

The high energy barrier observed in this Li^+^ penetration barrier likely arises for two reasons. First, the CHT molecule must undergo a significant deformation to accommodate for the cation penetration. The average C-C bond length in the initial Li-CHT-I complex is about 1.44 Å, increasing to 1.52 Å in the transition structure as the C-C bonds stretch to enlarge the ring opening, before contracting to 1.43 Å in the final CHT-Li-I structure. The second reason is the C-I bond which forms in the Li-CHT-I complex visible in Figure 10a. The bonded I anion is significantly less negative than an unbonded I anion, as shown in Table 5. As a result, this bonded I anion attracts the Li cation less, thus reducing the likelihood of Li penetration. Furthermore, as penetration occurs, this bond gradually weakens, as indicated by Figure 10b,c, where the sp^3^ hybridization of the bonded carbon atom transitions to a sp^2^ hybridization in the final product. This change in hybridization could increase the energy barrier.

### 2.5. Cation-Induced C-I Bonding

A predicted mechanism for C-I bond formation, which has not been previously described, was observed in the Li-CHT-I and Na-CHT-I insertion complexes (Figure 5). Typically, the I atom interacts with a carbon ring through substitution reactions, where the I atom replaces a H atom. In this case, however, the C-I bond forms without any substitution, resulting in an extra electron shared by the bonding carbon, which already had a filled valence. To accommodate this the C atom transitions from sp^2^ to sp^3^ hybridization. This change in hybridization is evident from the change in average bond angles around the C7 atom in Li-CHT-I (Figure 5b), which decreases to 109.32°, from 119.74° in the free CHT molecule. Similarly, the average bond angles around the C6 atom in Na-CHT-I (Figure 5d) decreases to 109.07°. In both Li-CHT-I and Na-CHT-I, the average bond angles are very close to the sp^3^ tetrahedral angles of 109.5°, whereas the original angles in the free CHT molecule closely align with the 120° angles between sp^2^ bonds. The C-I bond distance was found to be 2.30 Å in Li-CHT-I and 2.43 Å in Na-CHT-I. These distances, along with the change in carbon hybridization, suggest that this is a weak covalent interaction.

The formation of this bond causes the I anion to lose much of its negative charge and this extra charge is shared amongst the carbon atoms on the ring, evident by the less negatively charged I atoms and more negatively charged CHT molecule in Li-CHT-I and Na-CHT-I complexes when compared to the I anions and molecules of the other complexes (Table 5).

**Figure 11:**
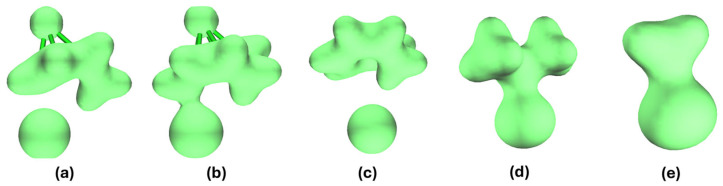
Calculated electron densities of (**a**) Na-NCD-I, (**b**) Na-CHT-I, (**c**) (CHT-I)^−^, (**d**) C_3_H_7_I, and (**e**) CH_3_I; isosurface value at 0.05 a.u.

The electron density plots (Figure 11) of the Na-CHT-I insertion complex clearly indicate the presence of this C-I bond, with the electron density visibly shared between the I and C atoms. In the electron density plot of Na-NCD-I where the C-I bond does not form, no electron density sharing between the I and C atoms is observed. This C-I bond in Li-CHT-I and Na-CHT-I can also be compared to the known C-I bonds, such as those in C_3_H_7_I and CH_3_I (Figure 11d,e). Notably, the electron density plot of the C-I bond in Na-CHT-I closely resembles the electron density plots of these two well-characterized bonds.

When no cation is present, as in the (CHT-I)^-^ binary complex (Figure 11c), the C-I bond is absent, suggesting that this bond is likely catalyzed by the presence of the Li and Na cations, hence the term cation-induced C-I bonding. In the bonding scenarios, the Li and Na cations attract the I anion in the insertion complexes, pulling it closer to the molecule to a point where the C-I bond formation becomes energetically favorable. The C=C double bonds in the ring compete with this C-I bond. If the I anion is not pulled sufficiently close, as in the Li-NCD-I, Na-NCD-I, K-NCD-I, and K-CHT-I insertion complexes, the double bond is favored over this C-I bond, and this bond will not form. Specifically, this bond is absent in the K-CHT-I complex because the larger size of the K cation prevents it from approaching the molecule as closely as the smaller Li and Na cations can, reducing the attraction between the K cation and the I anion and increasing the K-I distance. This increased K-I distance places the I anion farther away from the molecule, favoring the double bond over the formation of the C-I bond.

## 3. Methods

Ab initio calculations at the MP2 level of theory for geometry optimization and vibrational frequencies were performed using the NWChem package [21]. All calculations were performed using a Restricted Hartree-Fock (RHF) reference, as all studied systems are closed-shell species. The basis sets used were the incorporated aug-cc-pVDZ basis sets for light atoms and Stuttgart’s RLC effective core potentials for K and I, where the core replaced 18 and 46 electrons, respectively [22,23]. This level of theory provides accurate approximations without having to compromise for excessive computation power, as seen in Table 6. Most of the differences between the MP2 values and the experimental values are fairly consistent with previous studies, being less than 10% for dissociation energy (De), 3% for equilibrium distance (R_e_), and 8% for dipole moment (μ) [7]. The few exceptions observed can be attributed to the small absolute values, where although a large percent deviation is observed, the absolute deviations are not significant and ultimately fall under an acceptable range for the purposes of this study. Full unconstrained optimizations were carried out for all systems in the gas phase and vibrational frequencies were used to confirm energy minima. No solvent models were employed in this study, and all virtual orbitals were included in the calculations. Reaction barriers were obtained from a sequence of optimizations with geometry shifts along the appropriate coordinates, with other coordinates relaxed. Dipole moments were calculated as wave-function expectation values using the relaxed MP2 electron density. Charge analyses were done using atomic dipole moment corrected Hirshfeld charge analysis (ADCH) [24] using the GAMESS and Multiwfn programs [25,26].

## 4. Conclusions

Insertion complexes of NCD (norcaradiene) and CHT (cycloheptatriene) trapped between Li-I, Na-I, and K-I counterions were identified. The formation pathways of Na-NCD-I and Na-CHT-I were shown, where the stepwise attachment of ions was exothermic in both cases. Once formed, the metastable Na-mol-I insertion complexes are stabilized by the sizable ion recombination barriers (≥0.22 eV). These insertion complexes exhibit very large dipole moments due to their large charge separations, reaching up to 24.61 Debye for the K-CHT-I complex. The infrared spectra of all the insertion complexes were simulated, highlighting the differences between the insertion complexes, the free molecules, and the I-Na-Mol complexes, enabling the identification of these insertion complexes in future experimentation.

The insertion complexes were found to inhibit the NCD → CHT tautomerization, likely due to the combined effects of the strong field generated by the counterions and the pressure exerted on the molecule by the attraction between the counterions on either side. The Na-C_7_H_8_-I complex exhibited the strongest inhibition effect on the forward tautomerization, increasing the energy barrier from 0.03 eV to 0.11 eV. Conversely, the Li-C_7_H_8_-I complex most effectively inhibited the reverse CHT → NCD tautomerization, increasing the barrier from 0.26 eV to 0.47 eV. The K-C_7_H_8_-I complex increased the NCD → CHT tautomerization barrier while decreasing the reverse CHT → NCD barrier, resulting in this complex shifting the NCD ↔ CHT equilibrium towards the NCD side. This suggests that larger ions in such insertion complexes would likely increasingly push the equilibrium towards the NCD side. The above inhibition could be expected to be exhibited more efficiently at low temperatures.

## Figures and Tables

**Table 1:** Calculated geometry parameters of a-NCD and e-NCD compared to literature values.

Parameter ^3^	Lit [19] ^2^	a-NCD ^1^	e-NCD
R(C2C3)	1.507	1.509 [0.11%]	1.519
R(C2C5)	1.472	1.463 [−0.61%]	1.488
R(C5C7)	1.352	1.373 [1.52%]	1.382
R(C6C7)	1.454	1.450 [−0.25%]	1.473
∠(C2C3C1)	62.85	64.50 [2.63%]	60.48
∠(C3C2C5)	120.52	119.67 [−0.71%]	143.67
∠(C2C5C7)	122.00	121.94 [−0.05%]	110.45
∠(C5C7C6)	121.38	121.19 [−0.16%]	121.76

^1^ Percent difference shown in [brackets]; ^2^ B3LYP/6-31G* method used in the literature calculations; ^3^ Bond length in Å and bond angles in degrees.

**Table 2:** Calculated geometry parameters of CHT compared to experimental and theoretical literature values.

Parameter ^4^	Lit [19] ^2,3^	Exp [20]	CHT ^1,2^
R(C2C3)	1.509 {0.27%}	1.505	1.504 [−0.31%] {−0.05%}
R(C2C5)	1.351 {−0.37%}	1.356	1.372 [1.57%] {1.20%}
R(C5C7)	1.447 {0.07%}	1.446	1.443 [−0.28%] {−0.21%}
R(C6C7)	1.365 {0.66%}	1.356	1.388 [1.65%] {2.33%}
∠(C2C3C1)	108.3 {3.16%}	105.0	104.4 [−3.60%] {−0.55%}
∠(C3C2C5)	121.9 {0.11%}	121.8	120.1 [−1.49%] {−1.39%}
∠(C2C5C7)	125.4 {−1.39%}	127.2	124.3 [−0.93%] {−2.31%}
∠(C5C7C6)	125.9 {5.12%}	119.8	125.0 [−0.73%] {4.35%}

^1^ Percent difference compared to calculated literature values shown in [brackets]; ^2^ Percent difference compared to experimental literature values shown in {braces}; ^3^ B3LYP/6-31G* method used in the literature calculations; ^4^ Bond length in Å and bond angles in degrees.

**Table 3:** Calculated equilibrium parameters of the studied complexes (with M = Li, Na, K and H representing the H atom attached to C3 and pointing towards the molecule).

System	D_e_ ^1^	R_e_(M-C5) ^2^	R_e_(I-H) ^2^	R_e_(M-I) ^2^	μ ^3^
Li-NCD-I	5.79	2.13	2.60	4.92	12.93
Li-CHT-I	5.94	2.07	2.74	4.75	6.98
Na-NCD-I	4.82	2.51	2.61	5.38	16.01
Na-CHT-I	4.98	2.46	2.75	5.25	11.62
(Na-NCD)^+^	1.31	2.64	-	-	6.50
(NCD-I)^−^	0.40	-	2.59	-	8.67
(Na-CHT)^+^	1.32	2.63	-	-	6.40
(CHT-I)^−^	0.41	-	2.57	-	9.19
K-NCD-I	3.62	3.07	2.61	6.09	21.67
K-CHT-I	3.56	3.08	2.42	6.53	24.61

^1^ D_e_ relative to individual molecular fragments (ions + molecule) and is in eV; ^2^ R_e_ in Å; ^3^ μ in D.

**Table 4:** Equilibrium Distances of C1 and C2 atoms in NCD and CHT complexes.

System	R_e(C1C2)_ (NCD) ^1^	R_e(C1C2)_ (CHT) ^1^	ΔR_e(C1C2)_^1,2^
Free Mol	1.61	2.38	0.77
Li-Mol-I	1.63	2.40	0.77
Na-Mol-I	1.60	2.44	0.84
K-Mol-I	1.60	2.40	0.80

^1^ R_e_ distances in Å; ^2^ ΔR_e_ calculated as difference between the CHT and NCD R_e(C1-C2)_.

**Table 5:** Predicted charges of atoms in C_7_H_8_ systems.

	Li-NCD-I	Li-CHT-I	Na-NCD-I	Na-CHT-I	K-NCD-I	K-CHT-I
**Mol ^1^**	0.154	−0.157	0.042	−0.167	−0.041	−0.025
**M ^2^**	0.536	0.464	0.682	0.635	0.837	0.835
**I**	−0.690	−0.307	−0.724	−0.468	−0.796	−0.810

^1^ Mol refers to overall charge NCD/CHT molecule; ^2^ M refers to the cation in the complex (Li, Na, K).

**Table 6:** Equilibrium parameters of constituent atom-atom interactions calculated at the MP2/aug-cc-pVDZ level of theory.

System	De/eV [27] ^1^	R_e_/Å [28] ^1^	μ/D [28] ^1^
NaI	2.89 (3.15) [−8.36%]	2.79 (2.71) [2.73%]	9.70 (9.24) [4.99%]
LiI	3.42 (3.58) [−4.48%]	2.43 (2.39) [1.57%]	7.38 (7.43) [−0.68%]
HI	3.22 (3.09) [4.02%]	1.59 (1.61) [−1.04%]	0.30 (0.45) [−33.6%]

^1^ Experimental values shown in (parenthesis) and % difference shown in [brackets].

## Data Availability

The data presented in this study are available upon request from the corresponding author.

## Supplementary Materials

The following supporting information can be downloaded at: https://www.mdpi.com/article/10.3390/ijms27073086/s1.
