# Supplementary material for: Ion-Pair Mediated Valence Isomerization of Selected Cyclic C7H8 Molecules Trapped in Insertion Complexes
Source: Int J Mol Sci. 2026 Mar 28;27(7):3086. doi: 10.3390/ijms27073086 (PMC13072821; doi:10.3390/ijms27073086)
Supplement: Supplementary file 1 [file ijms-27-03086-s001.zip › ijms-4204176-supplementary.pdf]

Supplementary Materials: Field-Mediated Isomerization of Cyclic C_7H_8 Molecules Trapped in Ion-Pair Insertion Complexes

Chen Liang and Fedor Y. Naumkin *

Faculty of Science, Ontario Tech University/UOIT, Oshawa, ON, L1G 0C5, Canada; chen.liang@ontariotechu.ca

*Correspondence: Fedor.Naumkin@ontariotechu.ca

S1. Optimized Geometry Coordinates

Optimized XYZ coordinates, where the first column is the atomic number of the atom, and the following three columns are XYZ coordinates of the atom given in Å.

a-NCD

6	0.935559750	-0.805041360	0.419010010
6	0.935540650	0.805097360	0.418909800
6	1.586616500	-0.000003760	-0.678263950
6	-0.362811620	-1.435999930	0.181086140
6	-0.362822020	1.436019690	0.181089800
6	-1.441563230	-0.725181790	-0.282645680
6	-1.441563830	0.725223350	-0.282622890
1	1.611063230	-1.288183960	1.129037960
1	1.611060830	1.288273100	1.128906470
1	2.676665890	-0.000012150	-0.703828100
1	1.076199020	-0.000087550	-1.643910370
1	-0.470077030	-2.500283820	0.411971660
1	-0.470131350	2.500254840	0.411991740
1	-2.375883310	-1.249421630	-0.500667150
1	-2.375946410	1.249361210	-0.500551150

e-NCD

6	0.773527100	-0.708436000	-0.292332490

6	0.777298860	0.698302030	0.308200450
6	2.087269680	-0.014302300	0.019963870
6	-0.370215380	-1.439706890	0.315797280
6	-0.344804590	1.444669050	-0.321766590
6	-1.523320180	-0.682128600	0.237290900
6	-1.508811320	0.701591450	-0.268781330
1	0.609384610	-0.618765300	-1.379202690
1	0.591802890	0.611297570	1.391935300
1	2.665245210	-0.252508180	0.915375220
1	2.684658920	0.217324310	-0.864435930
1	-0.395817610	-2.498814310	0.594086170
1	-0.351221760	2.504537710	-0.598365090
1	-2.488532750	-1.124180940	0.504238790
1	-2.462414900	1.156398780	-0.555295490

CHT

6	0.962411520	-1.188770747	0.289307220
6	0.962362740	1.188839247	0.289460620
6	1.471065950	0.000097570	-0.479331190
6	-0.367981530	-1.521648480	0.241589000
6	-0.368105910	1.521530180	0.241801950
6	-1.391729850	-0.693874670	-0.348907090
6	-1.391746170	0.693691760	-0.348832470
1	1.658108010	-1.800159577	0.872966030
1	1.657990060	1.800238217	0.873176240
1	2.566616730	0.000104380	-0.552043090
1	1.032910100	0.000163230	-1.494357510
1	-0.688723870	-2.463219530	0.700483930
1	-0.688931630	2.463037880	0.700722150
1	-2.312604560	-1.188956480	-0.676294810

1	-2.312634720	1.188772000	-0.676122750

Na-NCD-I

6	1.091953580	-0.798956880	1.953324680
6	1.091882260	0.798945390	1.953373990
6	1.697845840	0.000052980	0.820504940
6	-0.220870570	-1.444660380	1.717386080
6	-0.220823230	1.444695700	1.717232060
6	-1.251400450	-0.731480610	1.135802890
6	-1.251396100	0.731411210	1.135876190
1	1.791441470	-1.290780100	2.636372990
1	1.791387280	1.290790770	2.636319900
1	2.785571900	-0.000005340	0.744799960
1	1.115203340	-0.000012530	-0.110049170
1	-0.288434380	-2.532968420	1.803373450
1	-0.288476420	2.533008680	1.803374380
1	-2.168300890	-1.239415870	0.832650420
1	-2.168291220	1.239362420	0.832635340
11	-0.854732450	-0.000005510	3.667630620
53	-0.950509160	-0.000055460	-1.712408380

Na-NCD

6	1.119220660	-0.844923610	0.030273290
6	1.119198480	0.844765970	0.030148280
6	1.742165370	0.000041890	-1.039909760
6	-0.184188700	-1.448840530	-0.215134260
6	-0.184320390	1.449171220	-0.215130610
6	-1.245032460	-0.724854370	-0.731616320
6	-1.244888760	0.724730180	-0.731570650
1	1.805279640	-1.334070730	0.726468400
1	1.805321980	1.334162550	0.726515280

1	2.831797300	0.000008910	-1.070381110
1	1.229367280	-0.000003510	-2.004471190
1	-0.303403900	-2.517323960	-0.006152310
1	-0.303413970	2.517120130	-0.006221480
1	-2.162848520	-1.247899950	-1.012909930
1	-2.162932930	1.247899960	-1.012951130
11	-0.891696790	0.000006930	1.879378360

NCD-I

6	1.486608370	-0.803567060	2.049395370
6	1.486503680	0.803609900	2.049388740
6	2.041643910	0.000110020	0.896940360
6	0.174022110	-1.435903080	1.903347120
6	0.173874520	1.435725060	1.903149590
6	-0.926717300	-0.724349510	1.500674130
6	-0.926741040	0.724035030	1.500389030
1	2.210293170	-1.283701440	2.715309670
1	2.210138980	1.283898900	2.715209100
1	3.129033580	-0.000059720	0.797436400
1	1.455792940	-0.000024280	-0.032857570
1	0.087217800	-2.508076370	2.108996120
1	0.087096440	2.508011880	2.108032810
1	-1.862996840	-1.249122410	1.293490850
1	-1.862984040	1.248712390	1.292801760
53	0.146810400	0.001085120	-2.263904710

Na*-I-NCD (*Refers to migrated ion, see Figure 6(a) of main text)

6	3.086788780	-0.809326710	0.310358330
6	2.918753480	0.844101750	0.363716800
6	4.118245540	0.154385510	-0.206282050
6	2.200596540	-1.489998790	-0.622690120

6	1.909309460	1.378101390	-0.538687500
6	1.509484210	-0.807523150	-1.607173050
6	1.370258160	0.634395770	-1.570642840
1	3.299281470	-1.281537040	1.272358460
1	2.970610850	1.265066320	1.369466710
1	5.035389440	0.225841480	0.378642270
1	4.230419130	0.195730530	-1.292463940
1	2.074821630	-2.573021260	-0.522425690
1	1.550580730	2.394162730	-0.349064550
1	0.926393170	-1.371946930	-2.340325690
1	0.678257140	1.109999120	-2.271047510
11	0.405829410	-0.215721000	0.946796610
53	0.765321790	1.143907080	3.372657710

Na-I*-NCD (*Refers to migrated ion, see Figure 6(b) of main text)

6	-2.587789650	-1.311449580	-0.720824630
6	-2.840778220	1.028859930	-0.918762400
6	-3.639147000	-0.242660960	-0.836285970
6	-1.913538590	-1.485018400	0.467755680
6	-2.240179140	1.536447150	0.212204010
6	-1.964606630	-0.560671860	1.575182200
6	-2.113816610	0.819298050	1.458455370
1	-2.390797840	-1.979159710	-1.565441770
1	-2.774969700	1.573430810	-1.865932070
1	-4.274250110	-0.385871300	-1.718844030
1	-4.261952880	-0.232833460	0.075868320
1	-1.283255960	-2.373879190	0.584636240
1	-1.815207830	2.545641370	0.168633780
1	-1.694823180	-0.948085920	2.562603980
1	-1.950228170	1.413589710	2.362846680

11	-0.098971690	0.169662240	-0.483563920
53	2.491569480	0.370225850	-1.415928320

Na-CHT-I

6	1.349723280	-0.888428751	1.451471555
6	1.386471428	1.534945954	1.701903598
6	1.956012889	0.380314884	0.903770949
6	0.036425535	-1.188842961	1.190033425
6	0.028532692	1.843800096	1.632677245
6	-0.912869390	-0.274466067	0.517357223
6	-1.022614491	1.058284218	1.065101894
1	1.974029252	-1.607393743	1.995114579
1	2.059990620	2.199801106	2.251737498
1	3.052366002	0.356214792	0.974592439
1	1.664637709	0.468274271	-0.156855852
1	-0.340078564	-2.180251108	1.476119018
1	-0.283437013	2.788633967	2.101245024
1	-1.891542346	-0.738415500	0.366838269
1	-1.974066956	1.573032910	0.899539357
11	-0.285140953	0.140066323	3.379386297
53	-0.340483464	-0.362925744	-1.846427165

Na-CHT

6	1.095628800	-1.189085870	-0.094026350
6	1.095502430	1.189194770	-0.093883680
6	1.606415340	0.000113330	-0.861365210
6	-0.239608280	-1.531029590	-0.160127920
6	-0.239747550	1.531003040	-0.159963610
6	-1.266597230	-0.696592410	-0.743391650
6	-1.266613700	0.696550950	-0.743298330
1	1.799001880	-1.832892330	0.444829850

1	1.798837360	1.832984120	0.445058290
1	2.700403980	0.000173480	-0.930811740
1	1.169041940	0.000164260	-1.875762310
1	-0.542084220	-2.514571400	0.219619130
1	-0.542316520	2.514497120	0.219920390
1	-2.178795570	-1.194523990	-1.086859330
1	-2.178924900	1.194451270	-1.086730460
11	-0.594553590	-0.000142500	1.945056910

CHT-I

6	1.279881750	-0.895504090	1.891443500
6	1.333672040	1.482285120	1.942175250
6	1.766007990	0.299999910	1.119630520
6	-0.058357800	-1.198233510	1.912178840
6	0.010515250	1.844250470	1.976656270
6	-1.089209660	-0.333029710	1.388608760
6	-1.057814220	1.049606370	1.417569150
1	1.989155240	-1.548747250	2.414401950
1	2.071241600	2.080175740	2.491439220
1	2.856688820	0.278069590	0.985739980
1	1.280715250	0.332628500	0.121031060
1	-0.375275610	-2.148849170	2.358341060
1	-0.263778570	2.788345800	2.463153310
1	-2.016131600	-0.803600980	1.043339160
1	-1.961849130	1.575923170	1.092751420
53	0.488879820	0.404931910	-2.318952790

Na*-I-CHT

6	1.777904790	-1.872067660	2.302830120
6	2.078177620	0.431464320	2.733661170
6	2.834129240	-0.866789420	2.669895550

6	1.295653880	-1.921016620	1.013458860
6	1.683828650	1.052910830	1.569278340
6	1.554620470	-0.917331300	0.009032870
6	1.732092540	0.440695000	0.262972290
1	1.425622760	-2.592820430	3.047339780
1	1.882322780	0.904131830	3.701274670
1	3.315678770	-1.108560000	3.625120970
1	3.593717750	-0.815753390	1.869614250
1	0.661341890	-2.767076340	0.725738270
1	1.294024620	2.075349600	1.629984540
1	1.431715830	-1.213836860	-1.037321930
1	1.735643830	1.110199670	-0.602836700
11	-0.587875340	-0.259698200	1.806640910
53	-3.287394430	-0.024043960	2.333928110

Li-NCD-I

6	1.508361620	-0.813249580	2.025464940
6	1.508342810	0.813364190	2.025497860
6	2.105299470	0.000016710	0.903568810
6	0.191030260	-1.449165880	1.796247950
6	0.191041840	1.449185630	1.796223200
6	-0.829503990	-0.729332810	1.191834930
6	-0.829519250	0.729334510	1.191831110
1	2.202726750	-1.296859580	2.717734830
1	2.202695460	1.296936860	2.717697120
1	3.192812550	0.000038250	0.823086830
1	1.521945930	0.000017320	-0.026765520
1	0.100437360	-2.530629720	1.925985320
1	0.100404430	2.530715840	1.925962190
1	-1.758781180	-1.228748040	0.913290230

1	-1.758813270	1.228777540	0.913270260
3	-0.232634470	0.000032520	3.299351430
53	-0.537240100	-0.000037520	-1.613943490

Li-CHT-I

6	1.311076510	-0.870276240	1.731102270
6	1.353015100	1.512099090	1.976547780
6	1.945814760	0.376510150	1.165577690
6	-0.000941200	-1.177684970	1.455628100
6	-0.008688520	1.836686900	1.877982480
6	-0.930109490	-0.280465930	0.710786720
6	-1.059302880	1.038861020	1.344622640
1	1.910022900	-1.556443970	2.340874030
1	2.017927910	2.186837320	2.522113090
1	3.037984950	0.346820520	1.278422770
1	1.697697020	0.470680680	0.097364900
1	-0.406541330	-2.130674990	1.817139920
1	-0.324276750	2.769380910	2.364690980
1	-1.915824640	-0.749714370	0.620416330
1	-2.040611290	1.516425200	1.272982560
3	-0.099111340	0.241209820	3.189945300
53	-0.397591000	-0.241356310	-1.525947520

K-NCD-I

6	1.585419960	-0.799403470	1.480110180
6	1.585393870	0.799396080	1.480025970
6	2.181580180	0.000037090	0.341644630
6	0.277822080	-1.444018680	1.250162020
6	0.277738540	1.444110880	1.250127180
6	-0.766649820	-0.729688870	0.709524140
6	-0.766662390	0.729742140	0.709512930

1	2.292174700	-1.289170990	2.158129420
1	2.292203030	1.289292330	2.158151320
1	3.269134160	0.000031940	0.262469770
1	1.590604070	0.000015750	-0.585930030
1	0.205563320	-2.531903130	1.354942930
1	0.205531420	2.531910370	1.354894360
1	-1.677108810	-1.241949800	0.391156890
1	-1.677092140	1.241934020	0.391146140
19	-0.637322330	0.000030270	3.802051990
53	-0.395278220	-0.000019080	-2.284451770

K-CHT-I

6	0.922575240	-1.071135170	1.477262150
6	0.909348250	1.318979370	1.785464950
6	1.548188850	0.208686600	0.991745280
6	-0.348820830	-1.365116190	1.049019900
6	-0.402859110	1.675037070	1.553830960
6	-1.223295920	-0.416145150	0.382908740
6	-1.317226200	0.936473000	0.715194150
1	1.509087610	-1.818737730	2.024205810
1	1.501380040	1.885668520	2.514784140
1	2.639271610	0.200213740	1.110926350
1	1.281385790	0.284617950	-0.084780680
1	-0.725468760	-2.388710910	1.178545080
1	-0.778053410	2.592228510	2.030057230
1	-2.037026300	-0.843137150	-0.205319350
1	-2.155283110	1.492321610	0.282132310
19	-1.462118200	-0.269114030	3.681670830
53	0.176368820	-0.802912440	-2.243838990

S2. Infrared Spectra

NCD

Frequency (cm^{-1})	Intensity ($D/Å^2$)
0	0
0	0.003
0	0
0	0.001
0	0.001
0	0.003
231.257	0.054
266.097	0.035
397.703	0.231
480.167	0.024
531.337	0.058
610.234	0
646.188	0.134
721.245	1.977
761.723	0.008
811.109	0.243
865.702	0.019
896.816	0.16
914.407	0.071
939.483	0
948.476	0.085
1009.928	0.07
1016.241	0.278
1047.77	0.108
1085.173	0
1093.142	0.012
1093.573	0.073
1175.14	0.009
1192.456	0.022
1218.719	0.037
1341.147	0.007
1387.017	0.074
1426.847	0.1
1458.814	0.134
1468.434	0.067
1575.591	0.065
1647.598	0.057
3162.839	0.213
3191.279	0.006

3198.358	0.121
3202.834	0.081
3209.323	0.406
3219.823	0.707
3230.574	0.325
3276.303	0.1

CHT

Frequency (cm^{-1})	Intensity ($D/Å^2$)
0	0
0	0
0	0
0	0
0	0
0	0
249.06	0.009
287.008	0.017
333.078	0.095
399.266	0.016
414.502	0.123
593.1	0.064
649.255	0.108
706.735	2.118
748.279	0.888
798.034	0.042
871.026	0.006
915.306	0.015
937.025	0.008
940.628	0.03
951.364	0.001
963.156	0.022
977.578	0.038
1046.49	0.033
1079.285	0.034
1177.297	0.012
1200.003	0.013
1220.888	0.002
1251.719	0.006
1288.756	0.064
1361.238	0.035
1405.424	0.075
1455.464	0.2
1465.802	0.001

1544.233	0.003
1622.264	0.012
1634.242	0.04
3039.748	0.594
3156.803	0.317
3177.409	0.001
3188.009	0.026
3191.783	0.039
3203.355	0.929
3212.025	0.753
3215.402	0.113

Na-NCD-I

Frequency (cm^{-1})	Intensity ($D/Å^2$)
0	0.136
0	0.097
0	0.091
0	0.003
0	0.009
0	0.168
60.535	0
89.988	0.604
107.366	1.166
145.262	0.776
204.528	0.115
257.98	0.147
297.733	0.131
323.897	1.106
460.557	0.062
464.822	0.018
539.127	0.235
567.652	1.092
648.396	1.584
735.992	0.098
746.711	3.068
806.24	0.364
868.582	1.151
882.278	0.151
892.147	3.348
910.686	0.263
933.273	0.015
976.362	0.031
1009.524	0.124

1054.133	0.022
1078.088	0.006
1102.521	0.143
1144.279	0.003
1162.524	0.369
1169.815	0.076
1207.746	0.094
1314.291	0.03
1368.692	0.094
1391.944	0.074
1432.153	0.113
1482.351	0.012
1551.66	4.307
1589.501	0.266
3084.278	2.105
3172.044	0.11
3180.814	1.009
3205.56	0.188
3207.557	0.015
3242.727	0.006
3253.6	0.001
3257.869	0.072

Na-CHT-I

Frequency (cm^{-1})	Intensity (D/Å^2)
0	0.096
0	0.098
0	0.049
0	0.051
0	0.005
0	0.003
75.121	0.489
100.629	0.286
123.37	0.078
152.761	2.37
209.922	0.586
225.969	0.922
249.869	0.265
333.92	0.644
351.688	1.762
432.409	0.79
529.049	0.983
553.781	3.146

639.418	0.766
697.336	0.429
721.192	1.676
753.586	0.538
856.929	0.094
883.448	5.425
893.187	0.672
927.084	0.741
933.015	0.126
961.536	0.109
1028.001	0.082
1072.487	0.356
1095.467	3.976
1177.466	0.464
1185.143	0.35
1222.528	0.173
1268.282	0.054
1300.183	0.13
1354.47	0.047
1392.082	0.317
1420.327	0.489
1464.589	0.528
1490.958	3.491
1562.708	4.081
1614.114	0.538
3050.87	0.637
3137.8	0.537
3146.281	0.164
3154.792	0.291
3184.596	0.478
3190.643	0.267
3200.356	0.244
3205.242	0.08

Na-I-NCD

Frequency (cm^{-1})	Intensity ($D/Å^2$)
0	0.002
0	0.062
0	0.016
0	0.024
0	0.022
0	0.032
36.151	0.146

57.892	0.268
79.201	0.026
123.758	0.09
174.194	0.312
214.95	0.15
263.865	0.685
281.559	0.083
345.081	0.219
468.288	0.008
493.831	0.067
617.725	0.003
644.842	0.089
733.568	2.675
780.117	0.072
829.205	0.222
868.635	0.102
898.33	0.151
913.675	0.137
944.392	0.05
947.409	0.071
1008.745	0.097
1018.411	0.299
1077.403	0.131
1084.716	0.095
1088.335	0.229
1101.196	0.016
1173.208	0.014
1187.959	0.076
1225.331	0.222
1339.789	0.033
1392.53	0.186
1430.159	0.171
1462.129	0.17
1472.88	0.155
1547.657	0.078
1621.143	0.039
3160.676	0.148
3194.745	0.006
3206.956	0.057
3213.008	0.095
3219.391	0.222
3222.804	0.223
3230.034	0.15
3277.209	0.003

Na-I-CHT

Frequency (cm^{-1})	Intensity ($D/Å^2$)
0	0.035
0	0.031
0	0.031
0	0.019
0	0.017
0	0.032
25.103	0.148
26.547	0.138
83.049	0.047
88.563	0.035
147.475	0.044
259.92	0.086
296.415	0.005
317.614	0.892
333.636	0.038
401.269	0.021
424.016	0.119
605.577	0.024
652.663	0.071
733.957	2.736
771.704	0.863
820.223	0.029
871.294	0.014
917.367	0.065
943.236	0.013
946.638	0.081
964.356	0.022
970.128	0
978.961	0.016
1049.385	0.038
1079.748	0.025
1182.513	0.012
1202.784	0.025
1227.219	0.001
1252.428	0.001
1291.485	0.122
1356.84	0.066
1402.364	0.122
1463.451	0.226
1465.886	0.008
1525.893	0.008
1609.091	0.011

1620.608	0.014
3053.393	0.34
3175.313	0.155
3188.362	0
3191.347	0.016
3202.458	0
3216.079	0.114
3216.228	0.092
3220.958	0.033

Li-NCD-I

Frequency (cm^{-1})	Intensity (D/$Å^2$)
0	0.149
0	0.079
0	0.077
0	0.008
0	0.005
0	0.118
43.97	0.01
115.669	0.463
134.156	1.939
262.149	0.601
290.041	0.017
336.947	1.026
415.707	0.569
421.161	0.58
474.288	0.018
478.476	0.042
546.627	0.115
554.632	2.353
644.647	0.82
751.136	0.067
754.329	4.58
815.927	0.377
874.211	1.01
882.382	0.121
901.917	2.849
923.042	0.254
934.741	0.005
984.718	0.057
1013.17	0.097
1061.208	0.02
1082.509	0.017

1099.26	0.213
1149.395	0.011
1169.133	0.343
1174.432	0.064
1210.44	0.116
1318.992	0.029
1375.419	0.135
1394.058	0.098
1435.631	0.214
1488.323	0.031
1539.433	4.192
1573.759	0.212
3080.546	2.128
3190.481	0.057
3197.098	0.664
3215.388	0.07
3217.513	0.019
3244.79	0.001
3255.498	0.026
3257.627	0.069

Li-CHT-I

Frequency (cm^{-1})	Intensity ($D/Å^2$)
0	0.045
0	0.001
0	0.002
0	0.033
0	0.005
0	0.067
87.903	0.222
146.847	0.028
193.228	1.177
235.401	0.199
283.844	1.277
304.858	0.034
371.437	1.291
387.931	0.246
445.595	0.531
472.013	1.91
542.406	0.696
579.998	2.288
662.569	0.37
708.816	0.735

716.919	1.619
757.512	0.512
855.896	0.104
883.107	4.916
890.616	0.33
920.37	0.219
931.338	0.448
955.254	0.223
1036.208	0.15
1071.151	0.339
1149.575	2.182
1175.426	0.289
1195.041	1.363
1225.035	0.072
1260.81	0.043
1296.397	0.175
1356.364	0.032
1383.326	0.94
1400.599	0.017
1469.725	1.015
1475.883	0.23
1569.118	3.698
1604.83	0.727
3077.762	0.589
3152.381	0.271
3155.843	0.135
3163.011	0.126
3174.712	0.064
3194.135	0.408
3205.607	0.12
3214.106	0.136

K-NCD-I

Frequency (cm^{-1})	Intensity (D/Å^2)
0	0.003
0	0.133
0	0.207
0	0.004
0	0.05
0	0.253
48.775	0.291
56.741	0.017
86.569	1.292

113.501	0.044
148.098	0.052
175.557	0.084
290.935	0.017
304.922	0.59
439.943	0.102
477.507	0.001
557.888	0.748
567.978	0.115
645.954	0.848
725.972	2.358
731.157	0.107
809.776	0.325
870.488	0.085
878.572	2.628
889.918	0.126
895.337	0.117
935.33	0.017
978.647	0.031
1010.95	0.141
1057.206	0.025
1078.755	0
1099.505	0.124
1150.932	0.008
1163.83	0.182
1178.84	0.041
1206.17	0.03
1321.685	0.025
1372.164	0.118
1401.635	0.074
1442.846	0.114
1481.987	0.023
1568.344	1.618
1614.561	0.169
3061.191	3.595
3161.475	0.144
3170.309	1.339
3190.627	0.403
3192.454	0.054
3231.287	0.06
3243.375	0.016
3254.139	0.177

K-CHT-I

Frequency (cm^{-1})	Intensity ($D/Å^2$)
0	0.162
0	0.125
0	0.128
0	0.003
0	0.198
0	0.039
46.673	0.638
51.543	0.023
70.341	1.305
104.505	0.003
143.892	0.119
163.649	0.128
240.985	0.026
301.397	0.701
339.934	0.171
406.498	0.351
460.493	0.049
552.472	0.522
655.971	0.459
701.446	1.017
749.323	0.85
778.372	0.456
865.253	0.31
903.869	1.812
909.569	0.743
926.636	0.195
946.389	0.262
952.446	0.019
979.839	0.075
1035.338	0.016
1072.731	0.086
1170.221	0.062
1186.865	0.012
1215.939	0.131
1272.579	0.271
1300.791	0.161
1353.548	0.064
1399.244	0.088
1451.461	0.128
1474.943	0.048
1527.594	2.318
1599.527	0.131
1614.211	0.145
2945.836	2.997

3142.088	0.338
3154.146	0.248
3160.721	0.414
3180.386	0.855
3189.259	0.598
3197.112	0.162
3245.374	0.047
